# Dysphagia Management and Stroke Units

**DOI:** 10.1007/s40141-016-0137-2

**Published:** 2016-11-23

**Authors:** David G. Smithard

**Affiliations:** 1 Department of Electronics and Digital Arts, University of Kent, Canterbury, UK; 2 Princess Royal University Hospital, King’s College Hospital, London, UK

**Keywords:** Dysphagia, Swallow, Swallowing disorders, Stroke, Stroke units, Review

## Abstract

Dysphagia is one of the many complications of stroke. It is common and is an independent marker of outcome. Dysphagia management is important. Although the speech and language pathologist is the key worker in dysphagia management, they are supported by all members of the multi-disciplinary team. Stroke patients should be screened on admission for the presence of dysphagia and assessed by the speech and language therapist (or appropriate professional), where indicated investigation should be undertaken to understand the swallowing physiology and to guide treatment. Management, at present, is based around texture modification of food/liquids and swallowing manoeuvres. Rehabilitation of swallowing remains in its infancy, but there is a lot of promising research with neurostimulation, medication and devices to strengthen muscles involved in swallowing.

## Introduction

Dysphagia is a common problem after stroke, with a reported prevalence up to 60%, which may rise to 100% if minor deficits such as minor tongue weakness are accepted as evidence of dysphagia. In many cases, dysphagia resolves fairly quickly, but in others, the swallow will vary in function [1, 2].

The act of swallowing is complex, not only peripherally but also centrally. Swallowing is essentially a reflex, which follows a set pattern initiated in the brainstem. The swallow is a synchronous and continuous event, once triggered. The events occur in a set order, but the duration of laryngeal elevation, UES opening and breath holding will vary depending on the bolus characteristics (volume and viscosity). Stroke affects swallowing at multiple levels due to the interruption of the feedback loop, with recovery depending on the cortical recovery [3].

With an ageing society, the incidence/prevalence of dysphagia is increasing. Many older people will have presbyphagia, and depending on their frailty, the prevalence of dysphagia may be as high as 70%; consequently, not all dysphagia on a stroke unit will be of a stroke origin. Work by Smithard et al. [1] found that there were people, who, 1 week after their stroke, had dysphagia which had not been noted at the time of their initial admission assessment. Swallowing after stroke is variable [1, 4]; therefore, constant awareness and review need to be undertaken to ensure that where problems exist, they are detected.

The identification and management of dysphagia are important to minimise the risk of infection (usually because of poor mouth care) [5, 6], distress due to aspiration of food and liquids and the ability to provide adequate calories.

## Stroke Units

Stroke management is multiprofessional and interprofessional not only in its entirety of the pathway but also sections of the pathway such as the management of swallowing problems. Good practice dictates that people admitted to hospital with an acute stroke should be managed on a stroke unit.

Stroke units are clinical areas dedicated to the care of people admitted to hospital with stroke. Stroke units are staffed by professionals with an interest in and knowledge of stroke. Evidence has shown that people cared for on stroke units recover better and are less likely to die both in the short and long terms (odds of death 0.82; 95% CI, 0.77 to 0.87; P0.00001) [7, 8].

It is not completely clear which elements of a stroke unit deliver the improved outcome. It is probably the overall ethos, coordinated care by expert staff, increased awareness engendered of stroke complications and care by such a unit, as well as the ability to be entered into clinical trials and to see a specialist [9–14].

Stroke team members should be conversant with dysphagia, how to identify it and what the management is, to position (sitting up between 45° and 90°, be alert and able to follow instructions), screen, assess and manage people with dysphagia. The inability to follow instructions does not mean that the assessment cannot be conducted; it does mean, however, that a novel or pragmatic approach may be required.

The clinical management of dysphagia will be complicated by the presence of hemianopia, sensory neglect, cognition and personal dislikes.

## Dysphagia Management

Dysphagia is defined as “difficulty in swallowing; the transfer of food from the mouth to the stomach”, whereas eating difficulties refer to the problems associated with the transfer of food/ liquid to the mouth. For many patients, the problems coexist.

The management of dysphagia is similar in most stroke services across the world, but is shaped by the availability of resources and the staff to undertake the service.

Many services are able to offer swallow screening at the time of admission or within 24 h by following a protocol. [15, 16]. There is much variability in the management plan, but managing stroke in a predetermined manner improves outcome [15, 17, 18]. The swallowing pathway has several key components to it. These are described in more details in various guideline documents [19] and a consensus statement by the ESSD [20•].

**Fig. 1:**
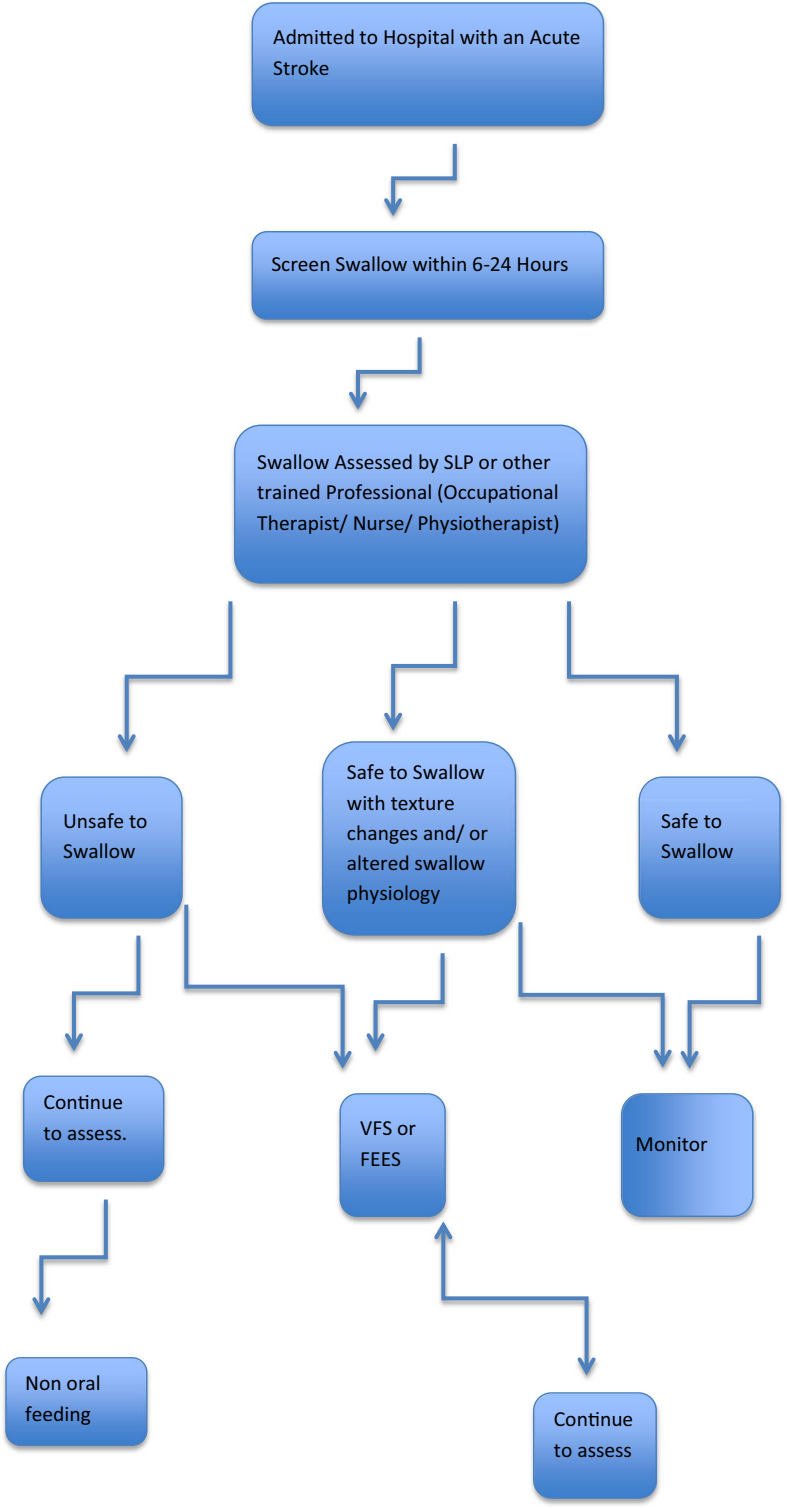
Swallow screening of patients admitted to hospital with an acute stroke

The generally accepted pathway consists of screening, assessment, investigation, management, rehabilitation and feeding (Fig. 1).

## Screening

There needs to be a consensus as to the purpose and meaning of screening. Within the acute stroke setting, it is generally taken to mean an assessment undertaken early, using one consistency (water) [21, 22]. Some screening tools, such as The Mann Assessment of Swallowing Ability (MASA) and miniMASA [23] rely on clinically generated variable to determine the presence of dysphagia and risk of aspiration. The screening of stroke patients for the presence of dysphagia is undertaken to identify those who can or cannot swallow safely. The swallow screen is not a diagnostic tool. Most swallow screens are based around the bedside swallowing assessment [1] and use varying volumes of water to assess the ability to swallow [1, 22, 23]. The swallow screen does not provide the ability to make swallowing management decisions beyond able to *swallow*/*not able to swallow* safely.

The question is which swallow screen and who undertakes it. There are many swallow screens available [24] therefore the need to develop, yet another similar screen is unwarranted. Who undertakes the screen will depend on the stroke service designed. In the UK, nurses from the stroke service will undertake the screen, usually on the ward; in the USA, Daniels reported on success of emergency department training nurses to undertake the screen [16, 25, 26].

**Fig. 2:**
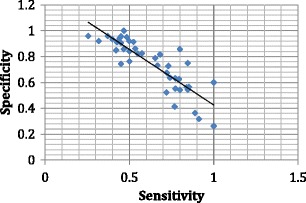
Relationship between specificity and sensitivity of swallow screens. Unpublished data (DG Smithard)

The sensitivities and specificities of swallow screens are varied (Fig. 2) and in attempts to improve the sensitivity of the water swallow screen, research had been undertaken on the additional benefit of cervical auscultation, arterial oxygen saturation and the addition of contrast media to the water supplemented with a chest radiograph [27]. Bours et al. [28] have suggested that oxygen saturation with the water swallow test is the best approach, yet the literature remains mixed [27]. Ramsey et al. [27] undertook a randomised study to investigate the added benefit of a chest radiograph to the bedside screen. Unfortunately, due to a mix of factors, recruitment of the appropriate patients was not possible [27].

A swallow screen needs to be sensitive to detect those with swallowing problems/aspiration and to those who have a normal swallow (specificity) (Fig. 2). These correlate reasonably well. The correlation between sensitivity and specificity and positive and negative predictive values, however, is generally poor (unpublished data). Kopey et al. [29] found that the sensitivity was poor but specificity was high in those with stroke that passed the test; 54.6% had clinically significant dysphagia. Their conclusion was that a high suspicion of dysphagia should remain in those with more dependent stroke (FIM <60). The swallow screen needs to be used easily and produce the same outcome independent on the operator [1, 26].

In Bristol, UK, a report at the European stroke conference suggested that the use of the miniMASA reduces the occurrence of aspiration pneumonia on the stroke unit by 75% (12 to 3%) [30]; however, recent reports suggest that now the study has finished, practice has returned to pre-study ways and chest infection rates are back at pre-study levels (personal communication).

Whichever screen is used and by whom may not be relevant, Hinchey et al. noted that the presence of a protocol in a service improved outcomes and reduced aspiration pneumonia [15, 31, 32].

The 2015 SSNAP [33] audit in the UK reported that nationally, 71% of swallows were assessed with a screen within 4 h of admission and 83.6% had an assessment by an SLT within 72 h. In Germany, figures were lower (2008) but screening occurred on 55.8–86.6% depending on the unit [34]. Titsworth et al. [35] found that using nursing staff to deliver a swallow screen followed by speech and language Pathologist (SLP) where indicated improved compliance with screening (39.3 to 72.4% *p* < 0.001) and consequent reduction in pneumonia.

## Swallow Assessment

Assessment is the next stage of management and involved a more detailed examination of the swallow, by someone trained in swallowing assessment [16, 36]. The assessment will review the oral anatomy, sitting balance and neurology of the patient as well as the swallow following stroke.

The swallowing assessment is conducted by those specifically trained to undertake it. The actual professional will vary by country [16, 20•, 36–42]. Whatever local situations dictate, the assessment should be early and undertaken by a trained specialist.

The swallow will be assessed using different consistencies of food/liquid (texture-modified food) and using different techniques to assist the swallow where indicated.

Following the swallowing assessment advice will be provided regarding the provision of food; this will range from normal diet, modified diet, speed of feeding, volume of each mouthful, need for supervision whilst eating/swallowing and the need for swallowing manoeuvres (e.g. head position, forceful swallow) or where the swallow is very unsafe, nil by mouth.

There is no standardised assessment [41], but the recent development of the V-VST [43] may provide a basis for a consistent approach.

A swallow assessed as safe may not remain safe during the length of someone’s stay in the stroke unit. The swallow may deteriorate with intercurrent events such as infection, uncontrolled diabetes, head injury following fall or recurrent stroke. Medication prescribed by the medical team may affect the swallow or result in confusion due to anticholinergic effects [44, 45].

## Investigation

Further investigation of the swallow may be required following clinical assessment. Any instrumental assessment needs to add value to the patient’s care; there are essentially two treatment methods, one radiological and the other endoscopic; they are complementary techniques [28, 46, 47].

The purpose of further assessment is to determine what is happening during the swallow. Videofluoroscopy (VFS) or modified barium swallow is often accepted as a “gold standard” for assessing the swallow. This is based on the fact that it was the first assessment developed. VFS provides data on bolus flow, muscle movement and the relationship of anatomy with the aspiration [2, 46]. However, it does entail the person being taken to radiology and a brief barium study and being exposed to x-rays. If someone is unable to sit in a special chair, the assessment may not be possible.

Fibreoptic endoscopic evaluation of swallowing (FEES) can be conducted at the bedside and can, therefore, be conducted in people who may be too unwell or who have poor sitting balance [48]. It does not expose people to radiology but requires a trained operator, and aspiration may be missed. FEES is not readily available in all units [16], though it is possible to train many different professionals in its use [49, 50].

Internationally, there are differences of opinion as to the clinical appropriateness of instrumental investigation; should all people undergo VFs or FEES or only where there is a clinical need to answer a specific question pertaining to management. [11–14]. Wilson in his MSc thesis [51] suggests that assessment followed by VF is the most cost-effective management structure; it is possible that VFS may delay the commencement of oral feeding and certainly, follow-up studies may not always show a physiological change despite a functional change.

## Rehabilitation

Rehabilitation and management of dysphagia are often confused. Many of the mechanisms used to assist people to swallow safely are thought of as rehabilitation techniques.

## Swallow Management

The provision of nutrients is important and fundamental; the management of dysphagia is to ensure the safe provision of adequate nutrition; failure to provide adequate nutrition will ultimately result in the death of the patient.

Presently, there are three approaches to managing a poor swallow: to alter the physiology of the swallow (manoeuvres) or to alter the consistency of the diet or a combination of both (Table 1). Research has shown that the use of texture modification and swallowing manoeuvres is beneficial in reducing aspiration and hopefully any consequences. However, there is a risk that the consistency of fluid thickening and food textures may not be consistent. Prepared foods/liquids are expensive. There are two groups working on the production of guidelines to assist [52•, 53•].

**Table 1:** Swallowing manoeuvres to assist in a reducing aspiration and producing a safe swallow

Forceful swallow
Double swallow
Breath holding
Supraglottic swallow
Suprsupra glottic swallow
Mendelssohn manoeuvre
Head turn
Chin tuck
Position of bolus in the mouth

All staff working on the stroke unit, the visitor (family/friend), volunteer, housekeeper, nurse, therapist and medical staff, need to be aware of any advice or instruction given by the SLP which should be visible and followed.

The SLP works in conjunction with the physiotherapist to assist with sitting and positioning, the occupational therapist to assist in special cutlery and plates/plate guards and the dietician to ensure adequate calories are being provided.

The greatest issue for patients and staff alike is the limited opportunity to undertake rehabilitation as opposed to the provision of assessment and advice. This is a result of a high workload and too few numbers of trained personnel. Despite the time constraints, dysphagia squeezes out dysphasia management in the acute and early post acute setting.

## Rehabilitation

Dysphagia rehabilitation as opposed to dysphagia management is gathering increasing momentum, with many researchers taking an interest and an increasing evidence base. The end outcome for any rehabilitation intervention is for someone to be able to swallow safely. Whichever technique is used, neuroplasticity will occur due to swallowing being a rich feedback system [54].

Physical rehabilitation of the swallow at present relies on the strengthening of hyoid musculature. This has taken several approaches, with variable effect. Tongue strengthening exercises [55–57] have been shown to benefit and improve swallowing safety after stroke. Exercises that work directly on the hyoid musculature for example the Shaker Manoeuvre [58] and more recently chin tuck against resistance (CTAR) [59•] also improve swallowing. Vitalstim [60] and Ampcare [61] both work via neuromuscular stimulation but more evidence is needed before either technique is accepted into everyday practice.

Scutt et al. [62] have been investigating the role of pharyngeal stimulation. A nasogastric tube, with electrodes and sensors, is passed to enable feeding and provide therapy by stimulating the pharynx at set thresholds and frequency for defined time periods. At present, further work is required to determine exactly who would benefit.

Transcranial magnetic stimulation has provided a great insight as to the recovery of swallowing, but as yet is not a rehabilitative technique available in the clinical setting.

## Nutrition and Hydration

As many as 24% of people admitted with an acute stroke are at risk of being malnourished [63]. More patients may become malnourished during admission due to many factors including illness, neglect, hemiparesis and hemianopia. Many people will require feeding and may not be supported [64, 65].

## Route

Many questions persist regarding the provision of food/nutrition as opposed to water. In the immediate 24–48 h of stroke, patients will need to be hydrated. Most guidelines suggest that an intravenous infusion of saline should be provided.

The optimal route for the provision of nutrition and hydration is orally or enterally. This keeps the gut functioning and reduces metabolic upset. If oral feeding cannot be provided, an alternative route can be considered. However, it has become recognised that those on a texture-modified diet have a reduced energy and protein intake and greater deficit to their requirements than those on a normal diet [66]; a similar situation is present for thickened fluids [67–69].

More contentious is the timing of the placement of a nasogastric tube. There are risks associated with nasogastric tube (NGT) placement including the placement of the tube within the bronchus of the right lung. Keeping NGTs in place can be difficult. NGTs frequently “fall out”. The reasons for this are multiple, from poor fixation to the patient pulling it out.

The use of NGT as a method of feeding is accepted. There has been scepticism as to the risk versus benefit of a nasogastric tube. An argument has been that the NGT may increase the risk of infection/aspiration, or cause physical damage to the pharynx, oesophagus or lung. NGTs can be misplaced and lead to feed passing into the lung if not spotted. Dzeiwas in 2003 [70] found that dysphagia was not exacerbated by the use of NGT and more recently Kalra et al. [71] have shown, in a larger study, that the use of NGT did not increase the occurrence of infection or mortality.

A small study by Davlos et al. [72] concluded that early enteral feeding did not prevent a negative protein balance. The FOOD [73] trial concluded that early feeding did reduce mortality by 5.8% but at the expense of increased disability in those that survived. Crary et al. [74] have not found a link between nutrition and dysphagia in the acute phase of stroke; the link with hydration is more complex as the more severe stroke patients will often be provided with intravenous hydration.

Consensus is that a nasogastric tube should be placed early to allow the administration of medication.

Following the FOOD trial [73], early placement of a percutaneous endoscopic gastrostomy tube was not recommended and could be delayed by several weeks, but this will be dictated as to whether the NGT remains in situ. Various devices have been used to maintain the NGT in place from tape, through the nasal bridle, to an American football helmet.

Appropriate identification of people requiring percutaneous endoscopic gastrostomy (PEG) feeding reduces the placement of tubes into people where it is not warranted, either because their care is palliative or that the swallow is improving.

Stroke units need to have policies and standard operating procedures in place, as to the timing of the provision of enteral nutrition. There are frequent delays between the decision to place the feeding tube to its placement and subsequent implementation. The placement of a PEG may vary due to the availability of services. Ideally, the PEG should be inserted within 72 h after the decision for insertion/placement has been made [14].

It is important not to procrastinate and excessively delay the provision of nutrition, as rehabilitation will be delayed, recovery is adversely affected.

Parenteral feeding should rarely be indicated following an acute stroke. In the majority of people, the gastrointestinal tract is functioning and therefore, enteral nutrition should be the route of choice. Where it is difficult to maintain NGT feeding or difficult to pass an NGT for anatomical reasons, parenteral nutrition is a useful interim route for nutrition, whilst waiting the placement of a PEG.

## Infection

The aspiration of food is potentially lethal, due to mechanical obstruction and pneumonitis [75, 76] rather than infection. The aspiration of saliva, on the other hand, carries a greater significant risk. The surface area of the teeth and gums is large [77, 78] and for every cubic centimetre, there are billion organisms. Mouth care is therefore vital but frequently neglected. Neglect is particularly a problem where non-oral feeding is being used, yet is relatively straight forward. Studies by Gosney et al. [79] and Rofes et al. [80] have shown that the use of simple mouth care with the addition of oral decontamination with metronidazole gel or mouthwash and toothpaste will reduce the incidence of infection [79, 80]. Latterly, Roffe’s group has shown that the use of metoclopramide may reduce the occurrence of aspiration [81].

The presence of hemiparesis and a reduction in chest wall movement on the hemiparetic side led to the theory that the lack of ventilation and sputum stasis may result in infection; however, two large randomised studies have shown that the use of prophylactic antibiotics did not reduce the occurrence of pneumonia in patients with dysphagia [82, 83].

## Future

Stroke will remain a burden on people, health services and society for many years. The incidence, if not prevalence, will increase as the population ages. Its management needs to be improved with rehabilitation occurring outside of hospitals. Research is required to develop devices that are simple, straightforward and at the same time effective. These devices need to be portable and to be able to be used by the patient alone or when being advised at a distance using telehealth.

Medication may aid those with an impaired rather than an absent swallow. Clavé’s group has been undertaking some interesting work with TRPV1 [83] receptor agonists; other works have suggested that Nifedipine, amantadine and substance P may offer some benefit [84, 85].

## Ethical Issues

Every stroke unit needs to be mindful that just because something can be done, it does not mean that it should be. Internationally, restraints are used to maintain the position of the NGT. In the UK, simple restraints are used regularly in an attempt to stop NGTs being removed. The use of mittens, physical restraints or chemical restraints raises not only legal but ethical issues.

People should not be compelled to undertake a course of action that may be unwarranted or unwanted. Both approaches could be perceived as assault, morally unacceptable and ethically undefendable if the treatment does not have any demonstrable likely benefit.

Stroke units need to have in place a protocol/policy, to manage dysphagia in patients where tube feeding is not warranted or not wanted. This will need to be developed in conjunction with end of life care.

## Conclusions

The management of dysphagia on stroke units is the concern of all who work there. Stroke units should have policies to direct the care and reduce the occurrence of pneumonia and other complications associated with aspiration.

The swallow should be promptly assessed and where indicated further investigations should be organised.

The institution of nutrition and hydration is of prime importance and needs to be implemented promptly.
